# Case identification of mental health and related problems in children and young people using the New Zealand Integrated Data Infrastructure

**DOI:** 10.1186/s12911-020-1057-8

**Published:** 2020-02-27

**Authors:** Nicholas Bowden, Sheree Gibb, Hiran Thabrew, Jesse Kokaua, Richard Audas, Sally Merry, Barry Taylor, Sarah E Hetrick

**Affiliations:** 1 A Better Start National Science Challenge, Auckland, New Zealand; 2 Department of Women’s and Children’s Health, University of Otago, 201 Great King St, Dunedin, 9016 New Zealand; 3 Department of Public Health, University of Otago Wellington, 23 Mein St, Newtown, Wellington, 6021 New Zealand; 4 Department of Psychological Medicine, University of Auckland, 22-30 Park Ave Grafton, Auckland, 1023 New Zealand; 5 Centre for Pacific Health, Va’a O Tautai, Health Sciences Division, University of Otago, 71 Frederick St, Dunedin, 9016 New Zealand; 6 Dean of the Otago Medical School, University of Otago, 290 Great King St, Dunedin, 9016 New Zealand

**Keywords:** Integrated data infrastructure, Administrative data, Big data, Mental health, Case identification

## Abstract

**Background:**

In a novel endeavour we aimed to develop a clinically relevant case identification method for use in research about the mental health of children and young people in New Zealand using the Integrated Data Infrastructure (IDI). The IDI is a linked individual-level database containing New Zealand government and survey microdata.

**Methods:**

We drew on diagnostic and pharmaceutical information contained within five secondary care service use and medication dispensing datasets to identify probable cases of mental health and related problems. A systematic classification and refinement of codes, including restrictions by age, was undertaken to assign cases into 13 different mental health problem categories. This process was carried out by a panel of eight specialists covering a diverse range of mental health disciplines (a clinical psychologist, four child and adolescent psychiatrists and three academic researchers in child and adolescent mental health). The case identification method was applied to the New Zealand youth estimated resident population for the 2014/15 fiscal year.

**Results:**

Over 82,000 unique individuals aged 0–24 with at least one specified mental health or related problem were identified using the case identification method for the 2014/15 fiscal year. The most prevalent mental health problem subgroups were emotional problems (31,266 individuals), substance problems (16,314), and disruptive behaviours (13,758). Overall, the pharmaceutical collection was the largest source of case identification data (59,862).

**Conclusion:**

This study demonstrates the value of utilising IDI data for mental health research. Although the method is yet to be fully validated, it moves beyond incidence rates based on single data sources, and provides directions for future use, including further linkage of data to the IDI.

## Background

Mental health problems are common among children and young people, with a worldwide estimated prevalence of 13.4% affected by any mental disorder [35]. In New Zealand, school-based survey results indicate 31% of young people experience at least two weeks of low mood, 15.7% report suicidal ideation, and 24% engage in self-harm each year [14]. The short-term consequences of childhood and adolescent mental health problems can include interference with education [38] and developmental milestones [16]. Longer term, they may be associated with personal costs, such as reduced employment [12, 31], poorer quality of life [6], and societal costs such as greater economic burden [39].

Most information regarding the prevalence and treatment of mental health problems originates from small cross-sectional studies with short-term evaluation, and occasional, expensive longitudinal studies with finite long-term outcomes. To date, there has been limited use of administrative data for mental health research [8, 19, 48], especially in children and young people [36]. However, large amounts of administrative data, including information on hospital attendance, community care specialist services, and medication prescriptions, are routinely collected and stored by national health providers and related institutions and may be valuable for health research [5, 17, 21]. Use of data for this purpose is permitted in some countries by privacy legislation [34].

The advantages of using administrative data for research include the large, heterogeneous and representative nature of samples, which allows the reflection of real-world populations and practice, ongoing tracking of problems via regular collection of up-to-date data, long observation periods and low cost. Disadvantages include erroneous interpretation of data beyond the scope for which they were intended, variability of data quality, limited clinical detail, and potential public concern about administrative data being used for research purposes [26]. In New Zealand, administrative data on most interactions with government service providers as well as a range of survey data are housed in Statistics New Zealand’s Integrated Data Infrastructure (IDI) [45]. The IDI is readily available, free to use, typically national in scope, and linked at the individual level.

Case identification for physical health problems using administrative data, typically utilising International Classification for Diseases (ICD) coding, has been widespread [1, 10, 20, 33, 37, 49, 50], but less so in mental health [15]. A standardised and accessible case identification approach means research can be more comparable, it negates the need to duplicate work, and permits researchers without specialist mental health knowledge to contribute more easily to the field. There are previous examples where New Zealand administrative mental health data have been used for case identification, but these are typically restricted to a narrow range of diagnoses and are not age specific [3, 23, 40].

This paper describes the development of an administrative data-based case identification method for research into the mental health of children and young people in New Zealand. The method utilises secondary care service use and medication dispensing data held within the IDI. Given that a large number of individuals with mental health problems are treated in primary care, through alternative therapies, or not at all, the method is not intended to calculate prevalence estimates for mental health problems but can be used to identify a relevant population for which we can examine health trajectories, comorbidities and a range of other wellbeing outcomes. These uses are especially important in understanding the burden of disease for mental health conditions and the impact they have on the lives of children and young people.

## Methods

### Integrated Data Infrastructure (IDI)

The IDI is a large research database managed by Statistics New Zealand, containing a wide range of administrative and survey data^1^ about people and households [45]. Cabinet directives dating back to 1997 mandated Statistics New Zealand to undertake cross-agency data integration and the IDI was established in 2011.^2^ Data in the IDI are held in a secure environment and can be accessed by approved researchers only for projects that are in the public interest. Since 1993 it has been possible to link health datasets together using the National Health Index (NHI) number, however, until the creation of the IDI, it was not possible to routinely combine health and non-health related data. The IDI provides secure access to linked data at an individual level. Data are linked probabilistically by Statistics New Zealand, usually using name, date of birth and sex.^3^ After linking, all identifying information is removed before the data are made available to researchers. The IDI enables more extensive use of government data for research, including supporting the evaluation of the long-term impact of health interventions, with the aim of improved public health [2].

### Data privacy

Statistics New Zealand’s ‘five safes’ framework [44] is used to ensure data privacy is protected. Only approved researchers can use the IDI for projects that have a statistical purpose and are for the public good. All data are de-identified and only accessible via a secure connection from a secure Datalab. Data and results must be aggregated and confidentialised according to Statistics New Zealand protocols [46], and all results are checked by Statistics New Zealand prior to their release from the secure environment.

The legal requirements to protect the IDI data include the Statistics Act 1975, the Privacy Act 1993, and the Tax Administration Act 1994 [44]. In addition to legal requirements a number of Statistics New Zealand policies, protocols, and guidelines exist [46]. These include guidelines for information privacy, security, confidentiality policy and data integration, microdata access, and privacy and confidentiality. Regular privacy impact assessments for the IDI also provide a systematic evaluation of the benefits and risks associated with integrating data from a number of sources [47].

### Data

Five datasets are used in this study. All are from administrative sources held within the IDI accessed in June 2019 using the most recent refresh of IDI data at that time. The datasets are each described below, including associated strengths and weaknesses for case identification of mental health and related problems.

### Programme for the integration of mental health data (PRIMHD)

PRIMHD is a national collection of publicly funded specialist mental health service use (PRIMHD activity data) and diagnoses (PRIMHD classification (diagnosis) data). Data are collected from district health boards (DHBs) and non-governmental organisations (NGOs) that provide specialist mental health services and used to report on which services are being provided, and who is providing the services for health consumers across New Zealand’s mental health sector [41]. A limitation of PRIMHD data is that it covers only publicly funded specialist mental health care which is targeted to the approximately 3 % of the population with the most serious mental health problems [27]. It does not cover mental health care provided in a primary care setting (the majority of mental health care in New Zealand), or mental health care provided in the private sector.

#### PRIMHD classification (diagnosis) data

PRIMHD classification (diagnosis) data in the IDI were collected from 1 July 2008 to 31 December 2016 and include primary and secondary diagnosis codes (ICD-10-AM and Diagnostic and Statistical Manual of Mental Disorders (DSM)-IV). PRIMHD is the only national collection of formal psychiatric diagnoses in New Zealand but it has some limitations. Some people have contact with mental health services but do not have specific diagnoses recorded in PRIMHD. For most (nearly 85% of these non-specific diagnosis codes), this is because clients were seen for only a brief period and there was insufficient time for a diagnosis to be assigned [29]. This gap in data is reflected in approximately 37% of all classifications (not clients).

#### PRIMHD activity data

PRIMHD activity data in the IDI were collected between 1 July 2008 and 30 June 2018 and contain a range of variables including (i) an activity type code, i.e. a code that classifies the type of healthcare provided, and/or (ii) a team type code, i.e. a code that identifies which team provided a service. These codes can be used to identify individuals with mental health problems. Using PRIMHD activity data, a diagnosis can sometimes be inferred from the type of service the client receives, as with substance use, and can help to improve the coverage and quality of diagnosis information.

### The National Minimum Dataset (NMDS)

NMDS is a national collection of publicly funded New Zealand hospital admissions, including day patients (stays of either 3 hours or more but not overnight) and emergency department visits of greater than 3 hours. Primary and secondary diagnosis codes (ICD-10-AM) are recorded for every hospital event and are used to identify mental health and related problems. NMDS data within the IDI were collected between 1 January 1988 and 31 December 2017. As there have been several changes to NMDS data collection over the years, most notably prior to 1994, for the current study NMDS use is restricted to 1994 onwards [42].

A key advantage of NMDS is the ability to utilise secondary diagnoses, i.e. issues deemed material to an individual’s care that are not the main reason for their hospital admission. For example, a patient may be admitted to hospital for a non-mental health reason but their mental health may affect treatment or recovery and is therefore recorded as a secondary diagnosis. In some cases, these individuals will not have accessed services for mental health, or may have done so only in the primary care setting (data not held in the IDI). In these circumstances, NMDS permits case identification of mental health problems not otherwise possible in the IDI.

### Socrates

Socrates is the national database of the Ministry of Health’s (MoH’s) Disability Support Services clients and service providers. Individuals have data recorded in Socrates when they apply for a needs assessment to access home help or other support services via a Needs Assessment and Service Co-ordination Agency (NASC) throughout New Zealand. A range of disabilities can be recorded on an individual’s record and these include some mental health diagnoses. These diagnoses come with the referral for the client; for example from a general practitioner, social worker, treatment and rehabilitation provider, or psychologist. Socrates was established in 2008 and data are robust from 1 January 2010. While Socrates data exist in the IDI prior to 2008 they are sparse and not deemed reliable.

There is uncertainty around the diagnostic detail in Socrates; it is not known who provides the diagnosis and therefore the accuracy of the diagnosis may vary. However, people with mental health problems, in particular neurodevelopmental disorders such as Attention Deficit and Hyperactivity Disorder (ADHD) can be identified. Some of these children and young people will not have been referred to specialist mental health services and therefore Socrates offers an additional source of case identifications.

### Pharmaceutical collection (pharms)

Pharms contains claim and payment information from pharmacists for government-subsidised medication dispensing throughout New Zealand [43]. Pharms data in the IDI were collected between 1 January 2005 and 31 December 2017.However, as pre-2007 data were collected with less than 90% coverage, only data collected from 2007 onwards were used for research purposes, as per MoH recommendations. ‘Chemical IDs’ assigned to each dispensing are used to identify the specific medication dispensed and can be used as indications for mental health problems.

The main advantage of pharms data is that they include information about both specialist and general practitioner prescribing. Therefore, pharms data provide some insights into mental healthcare activity at the primary care level. However, diagnostic information can only be inferred from pharmaceutical codes related to dispensing, therefore diagnoses derived from pharmaceutical data are less certain than those derived from PRIMHD and NMDS. They should be considered speculative because, while they provide information on dispensing of psychotropic medications, the use of any individual medication can be for a number of conditions. For the purposes of this paper, we have clustered data by the most likely use for a medication. At present, it is not possible within pharms to identify the health specialty of the prescriber (psychiatrist, general practitioner, or other medical practitioner) or the reason for the prescription [4].

### Mortality collection

The mortality collection contains information about the underlying causes of all deaths registered in New Zealand [28]. It uses the ICD-10-AM classification and *World Health Organization Rules and Guidelines for Mortality Coding* [51]. Mortality data are robust and of high quality. However, due to the timeframe for coronial processes, there is a two-year lag in its availability. For the current analysis, the mortality dataset within the IDI was collected between 1 January 1988 and 31 December 2015 and has been used in this study to identify cases of death by suicide.

### Data summary

**Fig. 1:**
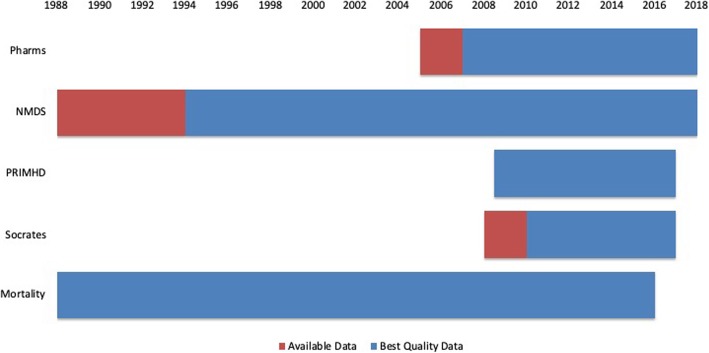
Dataset Coverage within the IDI

Figure 1 displays the periods of time^4^ each of the five datasets are available within the IDI. It breaks down the coverage by ‘available data’ and ‘best quality data’ as per the discussions above. Data were available for each of the datasets within a five-fiscal year period from 1 July 2010 (start of 2010/11 fiscal year) until 30 June 2015 (end of 2014/15 fiscal year). For the purposes of demonstrating an application of the method we decided to use the most recent of these fiscal years, 2014/15. The results are presented in the next section.

### Establishing and refining case definitions for mental health problems

Our aim was to create a method for identifying common, clinically relevant mental health problems for New Zealand children and young people (aged 24 and under) using available IDI data, which also included self-harm. The method built on a similar approach taken by the Social Investment Agency (SIA) [40] but was developed specifically for children and young people. The two-stage process undertaken to establish the case identification method is summarised below.

In the first stage, a short-list of 13 mental health (and related) problems of interest was derived by a clinically experienced team, based on the IDI data available. Our focus was on disorders that present to primary and secondary services. We were aware of the limitations of finer definition of problems because of the data available; for example, the sub-categories of anxiety disorder. For this reason, we chose broader categories that would allow us to make some limited assumptions about care in the primary setting that were sufficient for the purposes of population surveillance. Our final list comprised anxiety, depression, bipolar disorders, emotional problems (where anxiety and/or depression could not be reliably distinguished),^5^ disruptive behaviours, substance problems, eating problems, psychosis, personality disorders, sleep problems, self-harm, other mental health problems,^6^ and mental health not defined.^7^

The second stage of work involved the systematic classification and refinement of codes used to assign cases into each mental health problem category, by data source. A panel of eight specialists covering a diverse range of mental health disciplines (a clinical psychologist, four child and adolescent psychiatrists, two with dual qualifications as paediatricians, one with specific specialist expertise in substance-use disorders and three academic researchers in child and adolescent mental health) independently assigned diagnostic codes to the 13 mental health problem categories. Most data sources (NMDS, PRIMHD, Socrates, and the mortality collection) provided specific diagnoses (e.g. ICD-10-AM or DSM-IV) that fit naturally into the 13 mental health problem categories. For pharmaceutical data, the panel’s clinical expertise was utilised to infer the mental health problem according to the type of medication and patient age. Any disagreements were resolved by discussion and consensus. Five age strata (0–4, 5–9, 10–14, 15–19, and 20–24) were defined and used to increase accuracy of inferred diagnoses. These age bands were used as recommended management strategies depend on developmental level [24]. In addition, secondary mental health services are approximately organised around these age bands. Our method of classification drew on clinical experience and took into account the prevalence of disorder and the likely treatment within these age bands. For example, Amitriptyline is not included in the rubric for depression in children and adolescents but is for those aged 20 years and over. Medications deemed to be used for both anxiety and depression were assigned to the category ‘emotional disorders’ (e.g. Fluoxetine) and medications used for several mental health problems (e.g. Risperidone, which may be used for the treatment of psychosis, disruptive behaviour, obsessive compulsive disorder, bipolar disorder and emotional dysregulation) to the category ‘mental health not defined’. Medications considered more likely to be used for the treatment of non-mental health problems were entirely excluded. Details of all the individual codes used to determine each of the 13 mental health problem categories can be found in the Appendix.

The data sources used to identify each specific mental health problem group are outlined in Table 1. Some mental health problems were derived from as few as two datasets (e.g. eating problems were identified using NMDS and PRIHMD Diagnosis) and others from as many as four datasets (e.g. anxiety was identified using NMDS, PRIMHD, Pharms, and Socrates).

**Table 1:** Sources of data for each disorder group

Mental Health Problems	NMDS	PRIMHD	Pharms	Socrates	Mortality
Anxiety	✓	✓	✓	✓	
Depression	✓	✓	✓	✓	
Emotional Problems	✓	✓	✓		
Bipolar Disorders	✓	✓		✓	
Substance Problems	✓	✓	✓	✓	
Eating Problems	✓	✓			
Disruptive Behaviours	✓	✓	✓	✓	
Psychosis	✓	✓	✓	✓	
Personality Disorders	✓	✓		✓	
Sleep Problems	✓	✓	✓		
Self-harm	✓				✓
Other Mental Health	✓	✓			
Mental health not defined		✓	✓	✓	

### Data management

Data preparation was conducted in SAS 7.1 within the IDI environment. There were three main steps. First, event level data (e.g. medication dispensing for pharms, hospitalisations for NMDS) were extracted separately for each of the five datasets used in the study, for all individuals in the New Zealand youth population (0–24) for the 2014/15 fiscal year. Then, using the coding system for case identification described, 13 dichotomous mental health problem indicator variables were generated for each individual. Each dichotomous indicator was set to one if at least one code from the code list was found in any data source. Finally, data from each of the five datasets were appended and then collapsed to one set of mental health problem indicators per person. For individuals who had a ‘mental health not defined’ and another specific mental health problem group indicator (excluding self-harm) the ‘mental health not defined’ indicator was set to zero. The resulting data were analysed using StataMP 15. All counts were randomly rounded to base three in line with Statistics New Zealand confidentiality requirements.

### Establishing the New Zealand youth (0–24) population 2014/15

The New Zealand youth population (0–24) was calculated using existing methods for estimating a resident New Zealand population from the IDI [18, 52]. More specifically, this method included people whose presence in New Zealand was indicated by activity in key datasets. Individuals who had died^8^ or moved overseas were excluded. The total resident population generated using this method was within 2% of the official estimated resident population. Case identifications were restricted to people from within this population and 12-month prevalence rates were derived using this population as a denominator.

### Ethics approval

The University of Otago Human Research Ethics Committee reviewed the study for ethics consideration. The study was reviewed as a ‘Minimal Risk Health Research – Audit and Audit related Studies’ proposal and was approved. Approval to access IDI data was granted by Statistics New Zealand.

## Results

The case identification method was applied to data from the 2014/15 fiscal year. Over 82,000 unique individuals aged 0–24 with at least one mental health problem indicator including self-harm, other mental health, and mental health not defined were identified (see Table 2), indicating a 12-month prevalence of 5318 per 100,000 population (equivalent to 5.3%).

**Table 2:** Total Individuals by Disorder Group, Data Source and 12-month Population Prevalence Rates

Mental Health Problem	PRIMHD	NMDS	Pharms	Socrates	Mortality	Total	Pop. Rate^a^
Emotional	414	60	30,930			31,266	2020
Substance	14,022	3072	267	S		16,314	1742^b^
Disruptive	2772	177	12,414	591		13,758	889
Anxiety	3405	1377	6633	147		10,512	679
Depression	3288	1308	7116	21		10,392	671
Sleep	15	15	9549			9576	619
Psychosis	972	855	990	9		1881	201^b^
Eating	1092	297				1176	76
Personality	414	276		S		552	119^c^
Bipolar	327	210		S		420	65^d^
Sub Total (Any Problem)	**23,235**	**5715**	**54,417**	**693**	**n/a**	**71,229**	**4602**
MH not defined	8613		5445	S		10,101	653
Self-harm		2766			108	2877	186
Other MH	843	339				1149	74
Total (Any Problem)	**32,400**	**7629**	**59,862**	**693**	**108**	**82,296**	**5318**

^a^Total unique individuals (across all data sources) per 100,000 youth (0–24) population (unless otherwise stated)

S Data suppressed due to number of case identifications being less than 6

^b^per 100,000 10–24 year old population

^c^per 100,000 18–24 year old population

^d^per 100,000 15–24 year old population

The most prevalent mental health problem subgroups were ‘emotional problems’ with 31,266 individuals (2.0% of population), followed by substance problems with 16,314 individuals (1.7%), and disruptive behaviours with 13,758 individuals (0.9%).

Overall, pharms identified the greatest number of individuals (almost 60,000) and was also the data source used to identify the most individuals in six of the 13 problem groups (anxiety, depression, emotional problems, disruptive behaviours, psychosis, and sleep problems). PRIMHD was the data source that contributed the second most case identifications (over 32,000) and was the biggest contributor to a further six of 13 specific mental health problem groups (bipolar disorders, substance problems, eating problems, personality disorders, mental health not defined, and other mental health). NMDS was the only data source used to identify cases of non-fatal self-harm, and also contributed to the case identifications in all other mental health problem groups. Socrates was used in only eight of 13 mental health problem groups, however, while the corresponding case identifications numbers were generally low, it contributed to identifying nearly 600 disruptive behaviour cases.

## Discussion

### Key findings

We have proposed a method to identify and classify mental health problems among New Zealand children and young people using a range of data from the IDI. The method identified over 82,000 individuals aged between 0 and 24 with mental health problems, affecting 5.3% of all youth in 2014/15. Not surprisingly, emotional disorders, when combined with specifically defined anxiety and depressive disorders, comprise, by far, the greatest number of treated mental health problems. This is followed by substance problems.

The method is not designed to estimate prevalence of all diagnosed mental conditions due to an undercount arising from relying mostly on secondary service use data. However, it does provide a method for identifying a population of individuals with mental health problems at least serious enough to require some level of public health funded intervention. Additionally, it can provide information to help to understand the use of mental health services and pharmaceuticals, and more broadly facilitate research on those affected by mental health problems.

The results clearly demonstrate the value of utilising multiple datasets within the IDI, as there was no single dataset that performed well across all categories. The pharmaceutical collection data contributed the highest number of case identifications overall, however, in a number of mental health problem groups, other datasets were the main contributors, e.g. PRIMHD (substance) and NMDS (self-harm).

Having a method that can be used to simultaneously identify a range of mental health problems, including self-harm, at the individual level, and be able to link these data to other data sources (including non-health) could begin to set a framework to address important questions such as risk and protective factors, long term outcomes, health trajectories and burden of disease estimates for individuals with chronic mental health conditions.

### Limitations and strengths

A limitation of this method is the current lack of formal validation against other data sources. Formal validation would be useful for two reasons. First, it could establish whether the diagnoses recorded in administrative data and those inferred from pharmaceutical dispensing have been correctly assigned. This is typically done through a detailed review of medical or case notes [13, 25]. Second, validation could measure the level of undercount in the identified population, and the extent to which this undercount varies for different age, sex, ethnic, and other groups. One approach to measuring undercount is to compare our method against a dataset in which there is a complete record of mental health diagnoses, such as a sample survey or registry that contains complete information for a subset of the population [7, 32]. This may be possible in the future as the New Zealand Health Survey [30], which contains mental health information that may be useful for validation, is scheduled for inclusion in the IDI, but is not currently available. In the absence of a survey or other dataset containing complete mental health diagnosis information, statistical approaches such as capture-recapture may be useful to estimate the extent of undercount. These have been used previously with New Zealand administrative health data [22, 23], although they are not without challenges, in particular ensuring that the independence assumption is met [23].

The absence of primary care data means that people treated in primary care without medication (for example, those who are referred to brief intervention services or other publicly or privately funded psychological therapy) are not captured with existing datasets. Pharms data provides a way to account for people treated in primary care, however, it is the weakest dataset used in terms of clinical detail and accuracy. There is an increased risk of false positive case identifications when using pharmaceutical indications because some medications can be prescribed for non-mental health problems (e.g. amitriptyline for neuropathic pain). We have attempted to mitigate this risk by excluding medications that are considered to be used mostly for non-mental health problems, and imposing age restrictions on others to increase the likelihood that they are being used for mental health. However, until a formal validation process can be undertaken, the risk of over-identification remains and the assignment of diagnostic categories using medications should be considered an informed guess rather than a definitive classification.

The structure of the data sets and missing data from major and important sectors, such as the primary healthcare sector, means that this method should be considered with caution and treated as a first attempt to make sense of the national data. We have used a careful and transparent process to assign codes, with input from experts from a range of relevant backgrounds. Although multiple individuals were involved in assigning mental health codes to problem group categories for case identification, limited engagement was undertaken with clinical coders (those who ascribe diagnosis codes based on clinical records) and other clinicians and stakeholders, and this may have limited the accurate interpretation of data.

The case identification method described is based on administrative data that measures service use rather than prevalence of mental health problems. From epidemiological studies we know that for many common disorders such as depression and anxiety, the majority of young people do not access services. Therefore, the prevalence rates in this paper are likely to be lower than rates derived from surveys or other sources that are not based on service use. In addition, as evidenced by the large number of problems classified ‘not defined’, not all mental health problems can be classified using this method. Given that mental health problems are comprised of overlapping symptom clusters with often limited temporal stability, there may never be a perfect way to identify and track them using administrative data. Furthermore, administrative data lacks clinical detail and often has known quality issues, both of which may affect the accuracy of case identification.

The approach presented in this paper is not a panacea for mental health research in the IDI. Rather, it is an example of a broad approach that could be tailored by other researchers to suit the needs of their individual projects. For example, researchers may wish to exclude cases identified by medications if they want to minimise uncertainty. Furthermore, researchers should be aware of, and make explicit, the limitations of the method and contributing data sources. These limitations notwithstanding, the method provides a better means for identifying mental health problems than existing methods using single source service use data.

### Ethical issues

The secondary use of administrative data for research purposes is legal in New Zealand. The development of this administrative data into large linked data sources such as the IDI has raised issues around ethics and guidelines. Further discussion of these issues will be critical to the ongoing development and use of IDI data to ensure ethical use. The increased analytical power of such linked datasets needs to be balanced with the right to privacy for individuals, the lack of true informed consent, issues of data ownership in life and death, the veracity and completeness of available information, mechanisms for managing unexpected findings and agreed limits to the usage of data [11]. The possibility that continual comparison with other ethnic groups could disadvantage Māori and Pasifika people, who already face disparities in health, mental health and in a number of other areas, must be considered. Furthermore, that universal measures may not address the needs of specific cultural populations [9] should be borne in mind when applying data from this source.

### Further research and potential uses

Further research is needed to formally validate and potentially refine the described method. This may be undertaken initially using New Zealand Health Survey data that is scheduled to be uploaded into the IDI. Alternative approaches could include medical record review from either primary or secondary care data or capture-recapture methods. The development of a truly robust method is likely to be iterative and might include code weighting and further refinement of age restrictions or code allocations once a data source is available to validate against. Once validity has been demonstrated, the method could be used to track mental health problems in children and young people over time to better understand pathways to risk and resilience. The IDI method could also be used for evaluating the long-term impact of public mental health interventions and, in time, reducing health disparities and inequalities.

## Conclusion

We have described how multiple data sources from within the IDI can be used to identify and classify mental health problems according to secondary service use and medication dispensing data among New Zealand children and young people. This novel approach enables improved capabilities for mental health research and evaluation, however its current limitations should be kept firmly in mind. It could be further strengthened by the inclusion of additional data sources in the IDI, in particular primary care data. Undertaking a formal validation would allow for greater confidence in validity and also highlight areas where improvements can be made. The creation of the IDI is an important step forward in tracking health and well-being in New Zealand, but it is a new resource and ongoing work is needed to fully realise its potential for mental health research.

## Data Availability

The data used in this study are held with the Integrated Data Infrastructure and are managed by Statistics New Zealand. These data are publicly available, although access to is restricted. Please see https://www.stats.govt.nz/integrated-data/integrated-data-infrastructure/ for more details. The SAS code will be made available to interested parties.

## Appendix

**Table 3:** Codes Sets Used to Identify Mental Health Problem Groups

Mental Health Problem Group	NMDS (ICD-10-AM)	PRIMHD (DSM-IV plus ICD-10-AM codes listed under NMDS)	PRIMHD Team type / Activity type	Pharmaceuticals	Socrates
Anxiety	ICD-10-AM = F411, F4000^b^, F401^a^, F402, F408, F409, F931, F4001^b^, F410^b^, F412, F413, F418, F419, F064, F932, F420^a^, F421^a^, F422^a^, F428^a^,F429^a^, F450, F451, F452, F4530, F4531, F4532, F4533, F4534, F4535, F4538, F4539, F454, F458, F459, F480, F680, F681, F930, F430, F431, F432, F438, F439	DSM-IV = 30,002, 30,029, 30,022^b^, 30023^a^, 30,001^b^, 30,021^b^, 30,000, 29,384, 31,323, 3003^a^, 3007, 30,081, 30,082, 30,780, 30,789, 30,016, 30,019, 30,921, 3083, 30,981, 30,924, 30,928	None	Chemical ID = 6006, 1166, 1780, 2632, 1911, 1080^c^, 1730^b^, 1316^b^, 2636 (age 5–9 only)	Assigned diagnosis code = 1302
Depression	ICD-10-AM = F320^a^, F3200^a^, F3201%, F321^a^, F3210^a^, F3211%, F322^a^, F3220^a^, F3221%, F323^a^, F3230^a^, F3231%, F328^a^, F3280^a^, F3281%, F329^a^, F3290^a^, F3291%, F330%, F331%, F332%, F333%, F334%, F338%, F339%, F341%, F348%, F349%, F380^a^, F381%, F388^a^, F39^a^, F412, F251^a^, F0633	DSM-IV = 30,928, 29,620^a^, 29621^a^, 29622^a^, 29623^a^, 29624^a^, 29625^a^, 29626^a^, 29,630, 29,631, 29,632, 29,633, 29,634, 29,635, 29,636^c^, 29690^a^, 3004^a^, 3090^a^, 311^a^	None	Chemical ID = 1437, 1438, 3753, 1824, 2285, 2301, 3901, 1760, 2638, 1180, 3785, 1379^c^, 1642^d^, 1059^e^	Assigned diagnosis code = 1304
Emotional Problems	ICD-10-AM = F938, F939, F928, F929, F252^b^, F258^b^, F063, F0630, F0634, F0639, F920	DSM-IV = 3094, 3099, 29,383	None	Chemical ID = 2636^b^, 3926^b^, 3927^b^, 1030^b^, 1193^b^, 1190^b^, 1955, 6009, 1069, 1125^c^	None
Bipolar Disorders^c^	ICD-10-AM = F310, F311, F312, F313, F314, F315, F316, F317, F318, F319, F300, F301, F302, F308, F309, F340, F0631, F0632	DSM-IV = 29,600, 29,601, 29,602, 29,603, 29,604, 29,605, 29,606, 29,640, 29,641, 29,642, 29,643, 29,644, 29,645, 29,646, 29,650, 29,651, 29,652, 29,653, 29,654, 29,655, 29,656, 29,660, 29,661, 29,662, 29,663, 29,664, 29,665, 29,666, 2967, 29,680, 29,689, 30,113	None	None	Assigned diagnosis code = 1303
Substance Problems^b^	ICD-10-AM = F100, F101, F102, F103, F104, F105, F106, F107, F108, F109, F110, F111, F112, F113, F114, F115, F116, F117, F118, F119, F120, F121, F122, F123, F124, F125, F126, F127, F128, F129, F130, F131, F132, F133, F134, F135, F136, F137, F138, F139, F140, F141, F142, F143, F144, F145, F146, F147, F148, F149, F150, F151, F152, F153, F154, F155, F156, F157, F158, F159, F160, F161, F162, F163, F164, F165, F166, F167, F168, F169, F180, F181, F182, F183, F184, F185, F186, F187, F188, F189, F190, F191, F192, F193, F194, F195, F196, F197, F198, F199, F550, F551, F552, F553, F554, F555, F556, F558, F559	DSM-IV = 30,300, 30,500,30,390, 2910, 2913, 2915, 29,181, 29,189, 2919, 30,430, 30,520, 30,400, 30,550, 30,410, 30,540, 30,480, 30,490, 30,420, 30,560, 30,450, 30,530, 30,460, 30,590, 2920, 29,211, 29,212, 29,281, 29,284, 29,289, 2929, 30,440, 30,570	Activity type = T16, T17, T18, T19, T20, and/or Team type = 3	Chemical ID = 2367, 1432, 1841, 3950, 1795, 3793, 1252, 1273	Assigned diagnosis code = 1301
Eating Problems	ICD-10-AM = F500^b^, F501^b^, F502^b^, F503^b^, F508, F509, F982, F983	DSM-IV = 3071^b^, 30750^a^, 30,751^b^, 30,752, 30,753, 30,759	Team type = 16	None	None
Disruptive Behaviours	ICD-10-AM = F900, F908, F909, F901, F910, F911, F912, F918, F919, F920, F928, F929, F913, F631, F632, F638, F639	DSM-IV = 31,400, 31,401, 3149, 3093, 3094, 31,281, 31,282, 31,289, 3129, 31,381, 31,230, 31,232, 31,233, 31,234, V7102	None	Chemical ID = 3887^a^, 1809, 3880, 1389	Assigned diagnosis code = 1201, 1307
Psychosis^b^	ICD-10-AM = F200, F201, F202, F203, F204, F205, F206, F208, F209, F21, F220, F228, F229, F230, F231, F232, F233, F238, F239, F24, F250, F251, F252, F258, F259, F28, F29, F105, F115, F125, F135, F145, F155, F165, F175, F185, F195	DSM-IV = 29,510, 29,520, 29,530, 29,540, 29,560, 29,570, 29,590, 2971, 2973, 2988, 2989, 2913, 2915, 29,211, 29,212, 29,381, 29,382	Activity type = T09	Chemical ID = 3884, 1078, 1532, 2820, 1732, 1990, 1994, 2255, 2260, 1533, 1535, 1950, 3873,1007, 1226, 1283, 1583, 1799, 2298, 2530, 3803, 3898,3940, 4025, 8792	Assigned diagnosis code = 1306
Personality Disorders^e^	ICD-10-AM = F600, F601, F602, F6030, F6031, F604, F605, F606, F607, F608, F609, F61, F620, F621, F628, F629, F070	DSM-IV = 3010, 3019, 30,120, 30,122, 3014, 30,150, 3016, 3017, 30,181, 30,182, 30,183	None	None	None
Sleep Problems	ICD-10-AM = F510, F511, F512, F518, F519, F513, F514, F515	DSM-IV = 30,742, 30,744, 30,745, 347, 78,052, 78,059, 30,746, 30,747	None	Chemical ID = 3735, 2484^c^	None
Self-harm (self-harm also uses the ICD-10-AM codes listed under NMDS within mortality collection data)	ICD-10-AM = X60, X61, X62, X63, X64, X65, X66, X67, X68, X69, X70, X71, X72, X73, X74, X75, X76, X77, X78, X79, X80, X81, X82, X83, X84, Y870	None	None	None	None
Other Mental Health	ICD-10-AM = F050, F051, F058, F059, F060, F061, F062, F065, F066, F068, F069, F071, F072, F078, F079, F09, F488, F489, F633, F842, F843, F950, F951, F952, F958, F959, F980, F981, F984, F988, F989, F99, F630^b^	DSM-IV = 2930, 29,389, 2939, 29,910, 30,720, 30,721, 30,722, 30,723, 3073, 3076, 3077, 31,239, 31,382, 31,389, 3139, 78,009, 7876, 31,231^b^	None	None	None
Mental health not defined	None	DSM-IV = V7109, 3009, 7999	None	Chemical ID = 2466^c^, 3878^a^, 1315^a^, 1140^a^, 1183^a^, 1011^a^, 1729, 1731, 2295, 1397, 1865, 2224, 2436, 3892, 6007^c^	Assigned diagnosis code = 1399

^a^ Restricted to ages 5+

^b^ Restricted to ages 10+

^c^ Restricted to ages 15+

^d^ Restricted to ages 20+

^e^ Restricted to ages 18+

**Table 4:** Team Type and Activity Type Concordance (PRIMHD)

Team/Activity Type Code	Description
3	Alcohol and drug services
16	Eating Disorder Team
T09	Early psychosis intervention attendances
T16	Substance abuse Withdrawal management/detoxification occupied bed nights (medical)
T17	Substance abuse detoxification attendances (social)
T18	Methadone treatment specialist service attendances
T19	Methadone treatment specialist service attendances (consumers of authorised GP’s)
T20	Substance abuse residential service occupied bed nights

**Table 5:** Chemical Code Concordance (Pharmaceutical Collection)

Chemical Code	Description
1007	Sulpiride
1011	Risperidone
1030	Sertraline Hydrochloride
1059	Amitriptyline
1069	Amoxapine
1078	Clozapine
1080	Amylobarbitone sodium
1125	Nefazodone
1140	Olanzapine
1166	Bromazepam
1180	Venlafaxine
1183	Quetiapine
1190	Citalopram hydrobromide (Celapram)
1193	Citalopram
1226	Zuclopenthixol dihydrochloride
1252	Naltrexone hydrochloride
1273	Chlormethiazole edisylate
1283	Chlorpromazine hydrochloride
1315	Clomipramine hydrochloride
1316	Clonazepam
1379	Desipramine hydrochloride
1389	Dexamfetamine
1397	Diazepam
1432	Disulfiram
1437	Dosulepin (Dothiepin) hydrochloride
1438	Doxepin hydrochloride
1532	Flupenthixol decanoate
1533	Fluphenazine decanoate
1535	Fluphenazine hydrochloride
1583	Haloperidol
1642	Imipramine hydrochloride
1729	Loprazolam mesylate
1730	Lorazepam
1731	Lormetazepam
1732	Lozapine succinate
1760	Maprotiline hydrochloride
1780	Meprobamate
1795	Methadone hydrochloride
1799	Levomepromazine maleate
1809	methylphenidate hydrochloride
1824	Mianserin hydrochloride
1841	Naloxone hydrochloride
1865	Nitrazepam
1911	Oxazepam
1950	Pericyazine
1955	Phenelzine sulphate
1990	Pimozide
1994	Pipotiazine palmitate
2224	Temazepam
2255	Thioridazine hydrochloride
2260	Thiothixene
2285	Tranylcypromine sulphate
2295	Triazolam
2298	Trifluoperazine hydrochloride
2301	Trimipramine maleate
2367	Calcium carbimide
2436	Flunitrazepam
2466	Lithium Carbonate
2530	Haloperidol decanoate
2632	Alprazolam
2636	Fluoxetine
2636	Fluoxetine hydrochloride
2638	Moclobemide
2820	Fluspirilene
3753	Mirtazapine
3785	Venlafaxine
3793	Naltrexone hydrochloride
3803	Zuclopenthixol decanoate
3873	Ziprasidone
3878	Aripiprazole
3880	methylphenidate hydrochloride extended-release
3884	Amisulpride
3887	Atomoxetine
3892	Bupropion hydrochloride
3898	Zuclopenthixol hydrochloride
3901	Mirtazapine
3926	Escitalopram
3927	Sertraline
3940	Olanzapine pamoate monohydrate
3950	Buprenorphine with naloxone
4025	Paliperidone
6006	Buspirone hydrochloride
6007	Chlordiazepoxide hydrochloride
6009	Paroxetine hydrochloride
8792	Droperidol

**Table 6:** Assigned Diagnosis Code Concordance (Socrates)

Assigned Diagnosis Code	Description
1201	Attention deficit / hyperactivity, (e.g. ADD, ADHD)
1301	Alcohol / drug related disorder (excluding Korsakov’s syndrome)
1302	Anxiety disorders
1303	Bipolar disorder (manic depression)
1304	Depression
1307	Behavioural problem / issue, type not specified
1306	Schizophrenia
1399	Other psychiatric disorder (specific)

## Abbreviations

ADHD: Attention deficit and hyperactivity disorder
DHB: District health board
DSM: Diagnostic and Statistical Manual of Mental Disorders
ICD: International Classification for Diseases
MoH: Ministry of Health
NASC: Needs Assessment and Service Co-ordination Agency
NGO: Non-governmental organisation
NMDS: National Minimum Dataset
Pharms: Pharmaceutical collection
PRIMHD: Programme for the Integration of Mental Health Data
SIA: Social Investment Agency

## Footnotes

1 For more details on the data contained within the IDI see https://www.stats.govt.nz/integrated-data/integrated-data-infrastructure#data-in-idi

2 For more details on the history of the IDI development see http://archive.stats.govt.nz/browse_for_stats/snapshots-of-nz/integrated-data-infrastructure/idi-how-it-works.aspx#history

3 For more details on data linking within the IDI see http://archive.stats.govt.nz/browse_for_stats/snapshots-of-nz/integrated-data-infrastructure/idi-how-it-works.aspx

4 It is important to note that data in the IDI is updated periodically, typically quarterly.

5 This is a composite group formed because a number of medications exist which are typically good indications of either Anxiety Disorders or Depressive Disorders but not specifically one in particular. There are several diagnostic codes that contribute to this group as well.

6 This is a composite group, which for the sake of completeness includes all mental health diagnostic codes not otherwise used in the first ten groups.

7 This is a composite group formed because a number of medications are typically indications for a range of potential mental health problems but not specific disorders and in many cases. ‘Mental health not defined’ is also a diagnostic code commonly assigned to people with mental health problems that for whatever reason cannot be specified with more detail.

8 For fatal self-harm, the youth population for the previous fiscal year was used as the denominator to allow for mortality in the 2014/15 year.

## References

[1] Aronsky D, Haug PJ, Lagor C, Dean NC (2005). Accuracy of administrative data for identifying patients with pneumonia. Am J Med Qual.

[2] Atkinson J, Blakely T. New Zealand’s integrated data infrastructure (IDI): value to date and future opportunities. Int J Population Data Sci. 2017;1(1):105.

[3] Blakely TA, Collings SC, Atkinson J (2003). Unemployment and suicide. Evidence for a causal association?. J Epidemiol Community Health.

[4] Bowden N, Gibb S, Thabrew H, Audas R, Camp J, Taylor B, Hetrick S (2019). IDI trends in antidepressant dispensing to New Zealand children and young people between 2007/08 and 2015/16. N Z Med J.

[5] Cadarette SM, Wong L (2015). An introduction to health care administrative data. Can J Hosp Pharm.

[6] Chen H, Cohen P, Kasen S, Johnson JG, Berenson K, Gordon K (2006). Impact of adolescent mental disorders and physical illnesses on quality of life 17 years later. Arch Pediatr Adolesc Med.

[7] Dart AB, Martens PJ, Sellers EA, Brownell MD, Rigatto C, Dean HJ (2011). Validation of a pediatric diabetes case definition using administrative health data in Manitoba, Canada. Diabetes Care.

[8] Davis KA, Sudlow CL, Hotopf M (2016). Can mental health diagnoses in administrative data be used for research? A systematic review of the accuracy of routinely collected diagnoses. BMC Psychiatry.

[9] Durie M. Measuring Māori wellbeing. New Zealand Treasury Guest Lecture Ser. 2006;1:2007-09.

[10] Elixhauser A, Steiner C, Harris DR, Coffey RM (1998). Comorbidity measures for use with administrative data. Med Care.

[11] European Economic and Social Committee (2017). *The ethics of Big Data: Balancing economic benefits and ethical questions of Big Data in the EU policy context*.

[12] Fergusson DM, Boden JM, Horwood LJ (2007). Recurrence of major depression in adolescence and early adulthood, and later mental health, educational and economic outcomes. Br J Psychiatry.

[13] Fiest KM, Jette N, Quan H, St. Germaine-Smith C, Metcalfe A, Patten SB, Beck CA (2014). Systematic review and assessment of validated case definitions for depression in administrative data. BMC Psychiatry.

[14] Fleming TM, Clark T, Denny S, Bullen P, Crengle S, Peiris-John R (2014). Stability and change in the mental health of New Zealand secondary school students 2007–2012: results from the national adolescent health surveys. Aust New Zealand J Psychiatry.

[15] Frayne SM, Miller DR, Sharkansky EJ, Jackson VW, Wang F, Halanych JH (2010). Using administrative data to identify mental illness: what approach is best?. Am J Med Qual.

[16] Frisk M (1999). A complex background in children and adolescents with psychiatric disorders: developmental delay, dyslexia, heredity, slow cognitive processing and adverse social factors in a multifactorial entirety. Eur Child Adolesc Psychiatry.

[17] Garland A, Gershengorn HB, Marrie RA, Reider N, Wilcox ME (2015). A practical, global perspective on using administrative data to conduct intensive care unit research. Ann Am Thorac Soc.

[18] Gibb S, Bycroft C, Matheson-Dunning N (2016). Identifying the New Zealand resident population in the integrated data infrastructure (IDI). Statistics New Zealand.

[19] Harvey SB, Deady M, Wang M-J, Mykletun A, Butterworth P, Christensen H, Mitchell PB (2017). Is the prevalence of mental illness increasing in Australia? Evidence from national health surveys and administrative data, 2001–2014. Med J Aust.

[20] Hebert PL, Geiss LS, Tierney EF, Engelgau MM, Yawn BP, McBean AM (1999). Identifying persons with diabetes using Medicare claims data. Am J Med Qual.

[21] Hinds A, Lix LM, Smith M, Quan H, Sanmartin C (2016). Quality of administrative health databases in Canada: a scoping review. Can J Public Health.

[22] Jackson G, Wright C, Thornley S, Taylor WJ, Te Karu L, Gow PJ (2012). Potential unmet need for gout diagnosis and treatment: capture–recapture analysis of a national administrative dataset. Rheumatology.

[23] Kake T, Arnold R, Ellis P (2008). Estimating the prevalence of schizophrenia among New Zealand Maori: a capture–recapture approach. Aust N Z J Psychiatry.

[24] Kessler RC, Berglund P, Demler O, Jin R, Merikangas KR, Walters EE (2005). Lifetime prevalence and age-of-onset distributions of DSM-IV disorders in the National Comorbidity Survey Replication. Arch Gen Psychiatry.

[25] Kim HM, Smith EG, Stano CM, Ganoczy D, Zivin K, Walters H, Valenstein M (2012). Validation of key behaviourally based mental health diagnoses in administrative data: suicide attempt, alcohol abuse, illicit drug abuse and tobacco use. BMC Health Serv Res.

[26] Mazzali C, Duca P (2015). Use of administrative data in healthcare research. Intern Emerg Med.

[27] Ministry of Health. Looking forward: strategic directions for the mental health services. 1994. Retrieved from Wellington, NewZealand: https://www.moh.govt.nz/notebook/nbbooks.nsf/0/DAA659934A069A234C2565D70018A75A/$file/looking-forward.pdf.

[28] Ministry of Health (2017). Mortality collection data dictionary.

[29] Ministry of Health (2017). PRIMHD classification – summary and metadata.

[30] Ministry of Health (2019). New Zealand Health Survey.

[31] Mojtabai R, Stuart EA, Hwang I, Susukida R, Eaton WW, Sampson N, Kessler RC (2015). Long-term effects of mental disorders on employment in the National Comorbidity Survey ten-year follow-up. Soc Psychiatry Psychiatr Epidemiol.

[32] Muggah E, Graves E, Bennett C, Manuel DG (2013). Ascertainment of chronic diseases using population health data: a comparison of health administrative data and patient self-report. BMC Public Health.

[33] Neff JM, Sharp VL, Muldoon J, Graham J, Popalisky J, Gay JC (2002). Identifying and classifying children with chronic conditions using administrative data with the clinical risk group classification system. Ambul Pediatr.

[34] New Zealand Statutes (1993). Privacy act.

[35] Polanczyk GV, Salum GA, Sugaya LS, Caye A, Rohde LA (2015). Annual research review: a meta-analysis of the worldwide prevalence of mental disorders in children and adolescents. J Child Psychol Psychiatry.

[36] Reid G, Stewart SL, Zaric GS, Carter JR, Neufeld RW, Tobon JI (2015). Defining episodes of care in children’s mental health using administrative data. Adm Policy Ment Health Ment Health Serv Res.

[37] Saczynski JS, Andrade SE, Harrold LR, Tjia J, Cutrona SL, Dodd KS (2012). A systematic review of validated methods for identifying heart failure using administrative data. Pharmacoepidemiol Drug Saf.

[38] Schulte-Körne G (2016). Mental health problems in a school setting in children and adolescents. Deutsches Arzteblatt Int.

[39] Smith JP, Smith GC (2010). Long-term economic costs of psychological problems during childhood. Soc Sci Med.

[40] Social Investment Agency (2019). Using integrated data to understand mental health and addiction conditions.

[41] Statistics New Zealand (2015). IDI Data Dictionary : Programme for the integration of mental health data October 2015 edition.

[42] Statistics New Zealand (2015). IDI Data Dictionary : Publicly funded hospital discharges – event and diagnosis / procedure information November 2015 edition.

[43] Statistics New Zealand (2015). IDI Data Dictionary: Pharmaceutical data October 2015 edition.

[44] Statistics New Zealand (2017). How we keep IDI and LBD data safe.

[45] Statistics New Zealand. (2017b). Integrated Data Infrastructure. Retrieved from https://www.stats.govt.nz/integrated-data/integrated-data-infrastructure.

[46] Statistics New Zealand (2017). Legislation, policies, and protocols.

[47] Statistics New Zealand (2017). Privacy impact assessments for the IDI and LBD.

[48] Stewart R, Davis K (2016). ‘Big data’in mental health research: current status and emerging possibilities. Soc Psychiatry Psychiatr Epidemiol.

[49] Sung S-F, Hsieh C-Y, Lin H-J, Chen Y-W, Yang Y-HK, Li C-Y (2016). Validation of algorithms to identify stroke risk factors in patients with acute ischemic stroke, transient ischemic attack, or intracerebral hemorrhage in an administrative claims database. Int J Cardiol.

[50] Tu K, Campbell NR, Chen Z-L, Cauch-Dudek KJ, McAlister FA (2007). Accuracy of administrative databases in identifying patients with hypertension. Open Med.

[51] World Health Organisation (2003). Intentional self-harm.

[52] Zhao J, Gibb S, Jackson R, Mehta S, Exeter DJ (2017). Constructing whole of population cohorts for health and social research using the New Zealand integrated data infrastructure. Aust N Z J Public Health.

